# Lumbosacral transitional vertebra alters the mobility of the lumbar spine on flexion-extension radiographs

**DOI:** 10.1007/s00264-025-06637-7

**Published:** 2025-08-13

**Authors:** Anttoni Kuoppala, Juhani Määttä, Jaakko Hanhivaara, Jaakko Niinimäki, Mika Nevalainen

**Affiliations:** 1 University of Oulu, Oulu, Finland; 2 Oulu University Hospital, Oulu, Finland; 3 Medical Research Center Oulu, University of Oulu and Oulu University Hospital, Oulu, Finland

**Keywords:** Castellvi classification, Imaging, Lumbosacral, Mobility, Transitional vertebra

## Abstract

**Purpose:**

Lumbosacral transitional vertebra (LSTV) is a common anomaly linked to the degeneration of the lumbar spine. The aim of this work was to study lumbar spine mobility in subjects with and without LSTV using flexion-extension radiographs.

**Methods:**

In this retrospective single-center study, we identified subjects with flexion-extension radiographs and abdominopelvic CTs performed between years 2005–2023. LSTVs were graded according to Castellvi classification, and lumbar mobility evaluated through total lumbar lordosis, disc wedging angles, segmental lordosis angles, and range-of-motion (RoM) from the flexion-extension radiographs. Independent samples t-test and Mann-Whitney U-test were used for statistical analyses.

**Results:**

The study group comprised Castellvi types II-IV (*n* = 29, mean age 59.1 years, 62% males) and control group 20 subjects without LSTV (mean age 65.1 years, 35% males). The study group presented a smaller overall RoM of lumbar spine than controls (33.5°±14.2° vs. 38.3°±12.1°, *p* = 0.23). Distribution of total lumbar mobility differed in transitional L5/S1-level being 10.7% with study group and 22.2% with controls (*p* = 0.002); similarly, assessing disc wedging angles, extension and RoM were lower with study group than controls being 8.7 ± 4.8° vs. 12.9 ± 4.7° (*p* = 0.002) and 3.3 ± 3.8° vs. 7.3 ± 3.8° (*p* < 0.001), respectively. Same results were seen with segmental lordosis measurements: 15.7 ± 5.6° vs. 23.1 ± 4.5° (*p* < 0.001) and 3.3 ± 5.5° vs. 8.3 ± 3.8° (*p* < 0.001), respectively. There were no statistically significant differences of relative distribution of lumbar motion at the upper lumbar levels between the groups.

**Conclusions:**

LSTV decreases mobility of the lumbar spine in the L5/S1-level but does not increase relative motion at the upper lumbar levels. The overall compensation of mobility seems to distribute equally throughout the superior lumbar segments and not excessively to the superior adjacent level.

## Introduction

Lumbosacral transitional vertebra (LSTV) is a relatively common congenital anatomical variant located at the junction between the lumbar and sacral spine [1, 2]. In LSTV, the transverse processes of the lowest lumbar vertebrae are uni- or bilaterally enlarged and can be fused to the adjacent sacral ala completely with an osseous fusion or partially by a pseudoarticulation. Traditionally, anteroposterior (AP) radiographs of the lumbosacral spine have been used to identify subjects with LSTV [3]. This spinal anomaly is classified usually by the Castellvi radiographic classification which divides LSTV morphologically into four types (I-IV) [4]. Studies with large study populations have reported a prevalence ranging from 10 to 29% for LSTVs [2, 3, 5–8].

To date, there is still conflicting evidence between the association of LSTV and low back pain (LBP) [1, 8, 9]. LBP caused by LSTV is diagnosed as Bertolotti syndrome and it may originate from increased disc degeneration of the cranial adjacent disc to LSTV, extraforaminal stenosis, facet joint arthrosis or degeneration between the articulation of the LSTV and the sacral ala [10]. Intuitively, LSTV has an effect on the biomechanics and mobility of the lumbosacral spine, but this effect has not been studied extensively [8, 11]. Becker et al. (2022) [11] and Verhaegen et al. [12] reported that patients with LSTV have reduced range of motion (RoM) in the transitional segment (L5/S1) and increased RoM in the superjacent vertebral segment but the study populations were quite small. These different lumbar motion patterns and altered load transfers of the subjects with LSTV have been associated with increased disc degeneration (DD), facet degeneration (FD) and extraforaminal stenosis [7, 8, 13]. In this study we sought to evaluate the altered lumbar biomechanics caused by the LSTVs; accordingly, we studied the mobility of the lumbar spine using flexion-extension radiographs in subjects with and without LSTVs identified via CT imaging.

## Materials and methods

### Study population and data collection

We hypothesized that LSTVs alter the biomechanics of the lumbar spine by decreasing the mobility of the L5/S1 segment and increasing it on upper segments. Institutional review board approval was obtained and requirement for informed consent was waived for this single-center retrospective study. A picture archiving and communication system (PACS) search was conducted for subjects who had undergone a radiographic examination of the lumbosacral spine with flexion-extension radiographs between the years 2017 and 2023. Subsequently, corresponding abdominopelvic CT scans were searched from the PACS starting from the year 2005 and assessed for the presence of LSTV using the Castellvi classification. This initial assessment was performed by a medical student who was specifically trained for the task by a fellowship-trained musculoskeletal radiologist. The exclusion criteria for this study were: (1) an incomplete view of the entire lumbosacral spine, (2) spinal implantation in the lumbosacral spine, (3) immature skeletal development, and (4) bone tumour or acute fracture.

In the initial search we identified 57 subjects with LSTV which were matched for age with the control group consisting of 20 subjects without LSTV. As Castellvi type I does not represent a true lumbosacral transitional anatomy per se, we formed a study group (*n* = 29) comprising Castellvi types II-IV. We strove to have as short of a time period as possible between the flexion-extension radiographs and the CT scan. Accordingly, in situations with multiple flexion-extension radiographs, the examination closest to the date of the CT scan was chosen. On average the subjects had a little over two years (106 weeks) between the CT scans and flexion-extension radiographs: the biggest difference was approximately 13.5 years (703 weeks) and the smallest was 0 days meaning that the CT and lumbar flexion-extension radiograph was taken on the same day.

### LSTV classification

Transitional vertebrae were identified and categorized using the Castellvi radiographic classification, which divides LSTV morphologically into four types (I-IV). Types I-III can be also divided into unilateral (a) and bilateral (b) anomalies. In type I, the transverse process is dysplastic and measures over 19 mm in height. Type II includes pseudoarticulation between the transverse processes and the sacral ala. In type III, there is a complete osseous fusion between the enlarged transverse processes and the adjacent sacral ala. Type IV represents a combination of types II and III: an unilateral complete osseous fusion on one side and a pseudoarticulation on the contralateral side [4]. The classification was performed on the CT images by a fellowship-trained musculoskeletal radiologist with ten years of experience.

### Imaging techniques

#### Measurements

**Fig. 1:**
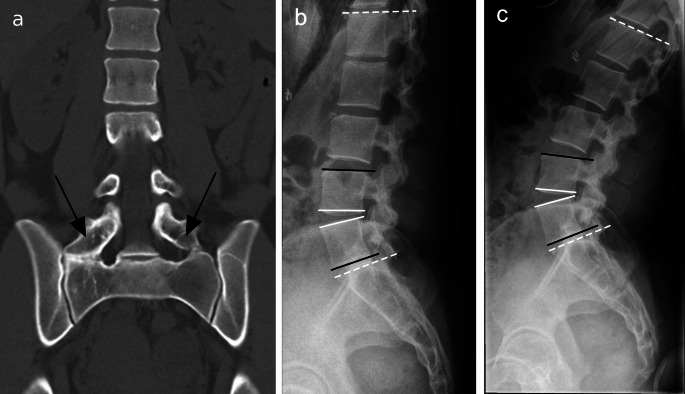
Example of a subject with lumbosacral transitional vertebra (LSTV) and measurements on flexion-extension radiographs: (**a**) shows a CT image of a subject with Castellvi IIb LSTV (black arrows points to the enlarged processus transversi). (**b**) depicts the flexion radiograph and (**c**) the extension radiograph and the performed measurements: the white lines illustrate segmental wedge angles and black lines segmental lordosis angles. The white dotted lines show the lumbar lordosis (LL) angle

**Fig. 2:**
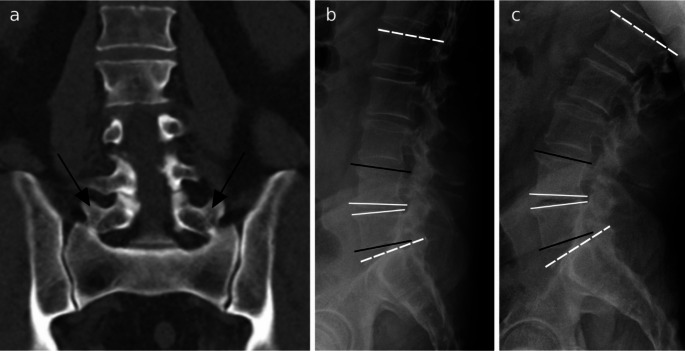
Example of a subject with lumbosacral transitional vertebra (LSTV) and measurements on flexion-extension radiographs: (**a**) shows a CT image of a subject with Castellvi IIb LSTV (black arrows points to the enlarged processus transversi). (**b**) depicts the flexion radiograph and (**c**) the extension radiograph and the performed measurements: the white lines illustrate segmental wedge angles and black lines segmental lordosis angles. The white dotted lines show the lumbar lordosis (LL) angle

We analyzed the effect of LSTV on lumbar motion patterns by measuring different angles of the lumbar spine (Fig. 1). The measurements of the parameters in this study were performed by a second year medical student under the guidance of the fellowship-trained musculoskeletal radiologist. Lumbar lordosis (LL) of the spine was estimated as the angle between L1 and S1 upper endplates. LL angles were measured from lateral ventral flexion and dorsal extension radiographs. The angle of LL was also measured from sagittal CT scans of the lumbosacral spine. In addition to LL angles, we measured segmental wedge angles and segmental lordosis angles. The angle between the lower endplate of the upper vertebral body and the upper endplate of the lower vertebral body was measured as the segmental wedge angle. Segmental lordosis angles were evaluated as the angle between the upper endplate of the upper vertebral body and the lower endplate of the lower vertebral body in every other segment but the lowest segment where this angle was measured between the upper endplate of the lowest lumbar vertebra and the upper endplate of S1. Figures 1 and 2 shows examples of the taken measurements.

### Statistical analysis

Statistical analyses were performed using the SPSS version 28.0 (IBM SPSS Statistics for Windows; Armonk NY; IBM Corp). Independent samples t-test and Mann-Whitney U-test were used to evaluate the differences between the groups.

Lumbar and segmental motions were analyzed both between controls and Castellvi types I-IV, and controls and Castellvi types II-IV, with our main focus with ‘true’ LSTV cases of Castellvi type II-IV. The level of statistical significance was set to *p* < 0.05. Total lumbar RoM was measured as a sum of all segmental wedge angles.

## Results

### Study population characteristics

Based on our initial search we identified 57 subjects with LSTV (mean age 64.8 years, range 28–87 years, 58% males): out of these, 28 (49%) were classified as type I, 17 (30%) as type II, nine (16%) as type III and three (5%) as type IV. Owing to the fact that Castellvi type I resembles normal lumbosacral anatomy, we performed our analyses with emphasis on the ‘true’ LSTV cases of Castellvi type II-IV. Accordingly, the final study group was formed from Castellvi types II-IV (*n* = 29, mean age 59.1 years, range 28–84 years, 62% males) and the control group from 20 subjects without LSTV (mean age 65.1 years, range 50–82 years, 35% males).

### Overall lumbar motion

The study group presented a slightly smaller, but statistically insignificant overall RoM of the lumbar spine than controls (33.5°±14.2° vs. 38.3°±12.1°, *p* = 0.23) (Table 1). The distribution of total lumbar mobility differed in the transitional L5/S1-level being 10.7% with LSTV and 22.2% with controls (*p* = 0.002); however, on the L4/5-level or upper lumbar levels no differences were seen, and the overall compensation of the mobility seemed to distribute equally throughout the superior lumbar levels (Table 2).

**Table 1:** Lumbar lordosis and range of motion in study and control groups

	Study group (Castellvi types II-IV) Mean (SD)	Control Mean (SD)	*p*-value
Lumbar lordosis [˚]	55.8 (± 9.3)	51.8 (± 10.0)	0.16
RoM flexion [˚]	29.9 (± 19.6)	22.2 (± 15.2)	0.15
RoM extension [˚]	63.4 (± 11.1)	60.5 (± 10.3)	0.32
Lumbar RoM [˚]	33.5 (± 14.2)	38.3 (± 12.1)	0.23

**Table 2:** Distribution of lumbar mobility to lumbar segments in study and control groups

	Study group (Castellvi types II-IV)	Control group	*p*-value
L5/S1 /Transitional segment	10.7%	22.2%	**0.002**
L4/5 /Cranial adjacent segment	23.8%	21.2%	0.38
L3/4	22.0%	19.2%	0.57
L2/3	22.4%	20.1%	0.16
L1/2	21.2%	17.3%	0.18

### Segmental mobility

When assessing the disc wedging angles of the L5/S1-level, the extension and RoM were lower with study group than controls being 8.7 ± 4.8° vs. 12.9 ± 4.7° (*p* = 0.002) and 3.3 ± 3.8° vs. 7.3 ± 3.8° (*p* < 0.001), respectively. Additionally, the flexion differed on L4/5-level being higher with the study group than controls 6.7 ± 4.3° vs. 3.2 ± 4.0° (*p* = 0.019). Similar results were seen with segmental lordosis measurements on L5/S1-level: the extension and RoM were lower with study group than controls with 15.7 ± 5.6° vs. 23.1 ± 4.5° (*p* < 0.001) and 3.3 ± 5.5° vs. 8.3 ± 3.8° (*p* < 0.001), respectively. On more cephalad segments, the study group showed increased mobility in the L2/3-level extension, and L1/2-level flexion and extension as compared to the controls (Table 3).

**Table 3:** Segmental lumbar lordosis range of motion in the study and control group

		Segmental wedge angle			Segmental lordosis angle	
	Study group (Castellvi types II-IV) mean (SD)	Control mean (SD)	*p*-value	Study group (Castellvi types II-IV) mean (SD)	Control mean (SD)	*p*-value
Flex. Trans. Seg./ L5/S1 [˚]	5.4 (± 3.2)	5.5 (± 3.1)	0.54	12.4 (± 5.7)	14.8 (± 4.8)	0.2
Ext. Trans. Seg./ L5/S1 [˚]	8.7 (± 4.8)	12.9 (± 4.7)	**0.002**	15.7 (± 5.6)	23.1 (± 4.5)	**< 0.001**
RoM. Trans. Seg./ L5/S1 [˚]	3.3 (± 3.8)	7.3 (± 3.8)	**< 0.001**	3.3 (± 5.5)	8.3 (± 3.8)	**< 0.001**
Flex. L4/5 [˚]	6.7 (± 4.3)	3.2 (± 4.0)	**0.019**	17.4 (± 8.7)	16.1 (± 6.7)	0.75
Ext. L4/5 [˚]	13.7 (± 6.4)	10.5 (± 4.9)	0.11	26.3 (± 8.7)	25.4 (± 5.6)	0.68
RoM L4/5 [˚]	7.0 (± 4.5)	7.3 (± 4.7)	0.81	8.9 (± 6.4)	9.3 (± 5.2)	0.72
Flex. L3/4 [˚]	5.3 (± 4.4)	3.8 (± 4.4)	0.29	9.3 (± 8.5)	8.4 (± 6.7)	0.84
Ext. L3/4 [˚]	11.5 (± 3.7)	10.5 (± 3.0)	0.27	17.9 (± 6.2)	17.2 (± 4.4)	0.51
RoM L3/4 [˚]	6.1 (± 3.4)	6.7 (± 4.0)	0.52	8.6 (± 5.4)	8.9 (± 5.1)	0.94
Flex. L2/3 [˚]	3.3 (± 4.0)	1.8 (± 3.4)	0.19	2.5 (± 7.3)	-0.2 (± 6.0)	0.11
Ext. L2/3 [˚]	10.2 (± 2.9)	8.7 (± 2.8)	0.08	11.5 (± 5.6)	8.8 (± 4.9)	**0.034**
RoM L2/3 [˚]	6.9 (± 3.9)	6.8 (± 2.8)	0.92	9.0 (± 4.6)	8.9 (± 3.2)	0.82
Flex. L1/2 [˚]	3.0 (± 2.6)	1.3 (± 2.9)	0.1	-2.0 (± 6.6)	-6.0 (± 4.7)	**0.03**
Ext. L1/2 [˚]	8.8 (± 3.5)	7.1 (± 2.9)	0.17	5.7 (± 6.2)	1.3 (± 3.9)	**0.006**
RoM L1/2 [˚]	5.8 (± 3.4)	5.8 (± 2.7)	0.87	7.7 (± 4.5)	7.2 (± 2.7)	0.67

## Discussion

In this study we evaluated the lumbar spine mobility in flexion-extension radiographs in subjects with transitional lumbosacral anatomy confirmed on CT scans. The study group consisting of Castellvi type II-IV subjects had reduced mobility at the transitional L5/S1 segment in comparison to the control group. Although transitional anatomy yielded a slightly poorer overall RoM of the lumbar spine, the difference was statistically insignificant. With the study group the distribution of total lumbar mobility differed being lower in L5/S1-level but the overall compensation of the mobility seemed to distribute equally throughout the superior lumbar segments and not excessively to the superjacent level.

To date there exist only two human studies of lumbar flexion-extension mobility in the setting of LSTV. In a retrospective study of 51 patients, Becker et al. (2022) reported that patients with LSTV had reduced mobility at L5/S1, and increased mobility at L1/2 and L2/3 in comparison to the control group. Although there was no statistically significant difference between the LSTV and control groups in the absolute mobility of the cranially adjacent segment L4/5, there was a significant difference in the distribution of lumbar mobility with 30.7% of overall lumbar mobility deriving from the L4/5 segment in the LSTV group vs. 21.6% in the control group [11]. More recently, Verhaegen et al. (2023) evaluated 13 LSTV cases against controls in standing and deep-seated flexion positions using radiographs. They reported that overall spinal flexion RoM with LSTV cases was like that in controls, but the restricted L5/S1 mobility was compensated in the most cephalad segments similar to our findings here [12]. However, both these studies had quite a small study population, did not differ Castellvi type I cases out of the analyses, and lacked a cross-sectional imaging to confirm the LSTV anatomy.

An in vitro cadaveric model study of Castellvi types IIa and IIIa also showed reduced mobility at L5/S1 and increased mobility at L2/3. In particular, lateral flexion and axial rotation were affected, but there was no difference between the groups in flexion or extension [14]. Nojiri et al. (2014) evaluated 15 subjects with LSTV using CT scans imaged in supine and 50° right rotation positions assessing segmental rotations and translations. In comparison to the control group, both pseudoarticulation and bony fusion resulted in greater rotation and translation in the cranial L4/5 segment, and lesser rotation and translation at the L5/S1 segment. There was no difference in mobility between pseudoarticulation and bony fusion sub-groups, and no hypermobility was observed in the upper lumbar spine [15]. We found no excessive relative motion at the superjacent level of LSTV and the overall compensation of the mobility seemed to distribute equally throughout the superior lumbar segments. This is contrary to the common hypothesis that especially the superjacent level of LSTV takes excessive load from the decreased LSTV level. We focused only on Castellvi II-IV LSTVs which may affect the results. LSTV has primarily innate nature [10] which may affect the mobility compared to operated fusion, for instance.

An association between lumbar degeneration and LSTV has been established in numerous studies with a distinct pattern to the degenerative changes. The transitional segment is mostly spared from degeneration while the cranial L4/5 segment is subject to increased stress and degeneration [7, 8, 13, 16–18]. This pattern of degeneration is akin to adjacent segment disease of spinal fusion patients [19]. It has been hypothesized that increased degeneration at the cranial segment is due to a compensatory hypermobility of said segment [13, 16, 20] but the evidence backing it up remains yet thin [11, 12, 15]. However, there are contradictory results between existing studies on how LSTV affects lumbar lordosis and the range of motion in the lumbar spine – in general, it appears that the transitional anatomy increases the lumbar lordosis [11, 12, 21–24]. In our study, we found no statistically significant difference in lumbar lordosis between the subjects with type II-IV LSTV and the controls. Our study group presented a slightly poorer overall RoM of the lumbar spine than controls but this difference was also statistically insignificant.

Some limitations exist within this study. First, this was a retrospective study where the sample remained rather low because CT confirmation of presence of true LSTV anatomy was insisted. Second, the flexion-extension radiographs were not fully standardized although a standing device reaching the pelvis level was used. Third, the time between the CT and radiographs was long but a necessity to collect a moderate subject sample. Fourth, we were not able to consider the possible effect of lumbar degeneration to the mobility of the spine. Fifth, the reproducibility of the lumbar measurements was not studied here.

In conclusion, the lumbosacral transitional anatomy decreases the mobility of the lumbar spine in the L5/S1-level but does not increase the relative motion at the adjacent or upper lumbar levels. Thus, due to the primarily innate nature of LSTV, it may not load the superjacent level as much as previously thought.

## Data Availability

No datasets were generated or analysed during the current study.
